# MRI-based clinical-radiomics model predicts tumor response before treatment in locally advanced rectal cancer

**DOI:** 10.1038/s41598-021-84816-3

**Published:** 2021-03-08

**Authors:** Andrea Delli Pizzi, Antonio Maria Chiarelli, Piero Chiacchiaretta, Martina d’Annibale, Pierpaolo Croce, Consuelo Rosa, Domenico Mastrodicasa, Stefano Trebeschi, Doenja Marina Johanna Lambregts, Daniele Caposiena, Francesco Lorenzo Serafini, Raffaella Basilico, Giulio Cocco, Pierluigi Di Sebastiano, Sebastiano Cinalli, Antonio Ferretti, Richard Geoffrey Wise, Domenico Genovesi, Regina G. H. Beets-Tan, Massimo Caulo

**Affiliations:** 1 Department of Neuroscience, Imaging and Clinical Sciences, “G. D’Annunzio” University, Via dei Vestini, 66100 Chieti, Italy; 2 Department of Radiation Oncology, SS. Annunziata Hospital, “G. D’Annunzio” University of Chieti, Via Dei Vestini, 66100 Chieti, Italy; 3 Department of Radiology, Stanford University School of Medicine, Stanford, CA USA; 4 Department of Radiology, Netherlands Cancer Institute, Amsterdam, The Netherlands; 5 Unit of Radiology, “San Pio da Pietralcina” Hospital, Vasto, Italy; 6 Unit of Ultrasound in Internal Medicine, Department of Medicine and Science of Aging, “G. D’Annunzio” University, Chieti, Italy; 7 Department of Innovative Technologies in Medicine and Odontoiatry, “G. D’Annunzio” University, Chieti, Italy; 8 Division of Pathology, ASST of Valtellina and Alto Lario, Sondrio, Italy; 9 GROW School for Oncology and Developmental Biology, Maastricht University, Maastricht, The Netherlands; 10 Department of Radiology, University of Southern Denmark, Odense, Denmark

**Keywords:** Cancer imaging, Cancer therapy, Tumour biomarkers

## Abstract

Neoadjuvant chemo-radiotherapy (CRT) followed by total mesorectal excision (TME) represents the standard treatment for patients with locally advanced (≥ T3 or N+) rectal cancer (LARC). Approximately 15% of patients with LARC shows a complete response after CRT. The use of pre-treatment MRI as predictive biomarker could help to increase the chance of organ preservation by tailoring the neoadjuvant treatment. We present a novel machine learning model combining pre-treatment MRI-based clinical and radiomic features for the early prediction of treatment response in LARC patients. MRI scans (3.0 T, T2-weighted) of 72 patients with LARC were included. Two readers independently segmented each tumor. Radiomic features were extracted from both the “tumor core” (TC) and the “tumor border” (TB). Partial least square (PLS) regression was used as the multivariate, machine learning, algorithm of choice and leave-one-out nested cross-validation was used to optimize hyperparameters of the PLS. The MRI-Based “clinical-radiomic” machine learning model properly predicted the treatment response (AUC = 0.793, *p* = 5.6 × 10^–5^). Importantly, the prediction improved when combining MRI-based clinical features and radiomic features, the latter extracted from both TC and TB. Prospective validation studies in randomized clinical trials are warranted to better define the role of radiomics in the development of rectal cancer precision medicine.

## Introduction

In recent years, neoadjuvant chemo-radiotherapy (CRT) followed by total mesorectal excision (TME) became the standard treatment for patients with locally advanced (≥ T3 or N+) rectal cancer (LARC)^1,2^. This approach increased the chance of a significant downstaging and reduced the risk of recurrence^3–6^. In this context, Magnetic Resonance Imaging (MRI), thanks to its diagnostic and prognostic relevance, is the gold standard imaging approach for local staging and treatment response assessment^7^. At staging, MRI provides information on the site, the extension and the relation with surrounding organs, thus establishing landmarks for following treatment. Moreover, it reveals prognostic signs such as the mesorectal fascia involvement, the extramural vascular invasion and the distance to the anal sphincter complex^8^. During the treatment and, mostly, at the end of the CRT right before surgery, MRI plays a crucial role for the response assessment^9–15^. In fact, approximately 15% of patients with LARC shows a complete response to CRT (complete responders) and it has been suggested that surgery could be omitted in selected patients^3^. Moreover, an international multicenter registry study on watch and wait (W&W) strategy revealed a 5-year disease specific survival of 94%^16,17^. In this context, the use of MR as predictive biomarker in oncology quickly became a hot topic in literature, with the primary aim to select the most appropriate treatment thus pursuing precision medicine^18^. Moreover, the development of organ preservation policies further encouraged the researchers to investigate new MRI-based biomarkers for treatment response^3^. This approach could be extremely beneficial for patients considered good responder, since the neoadjuvant treatment could be intensified to increase the chance of organ preservation. Moreover, for these patients, a longer interval between CRT and surgery (from 8 to 11 weeks) may increase the pathological complete response rates as well. On the other hand, in poor responders, the treatment could be tailored with an additional boost^19^. Methods extracting mineable data from clinical images, such as those based on automatic extraction of features from radiological images (radiomic features) and machine learning approaches, revealed promising results when combined to visual morphologic and clinical assessment^20–28^. Moreover, in other tumor types, prognostic information has been obtained not only from the tumor but also in the tumor surrounding tissue (TST)^29–31^. We hypothesized that a combination of MRI-based data, including clinical and computational ones, could be useful for treatment response prediction at an early stage.

We here present a novel machine learning model combining MRI-based clinical and radiomic features, the latter extracted from both the “tumor core” (TC) and the “tumor border” (TB) from baseline T2-weighted (T2w) images, for the early prediction of treatment response in patients with LARC.

## Methods

### Subjects

**Figure 1:**
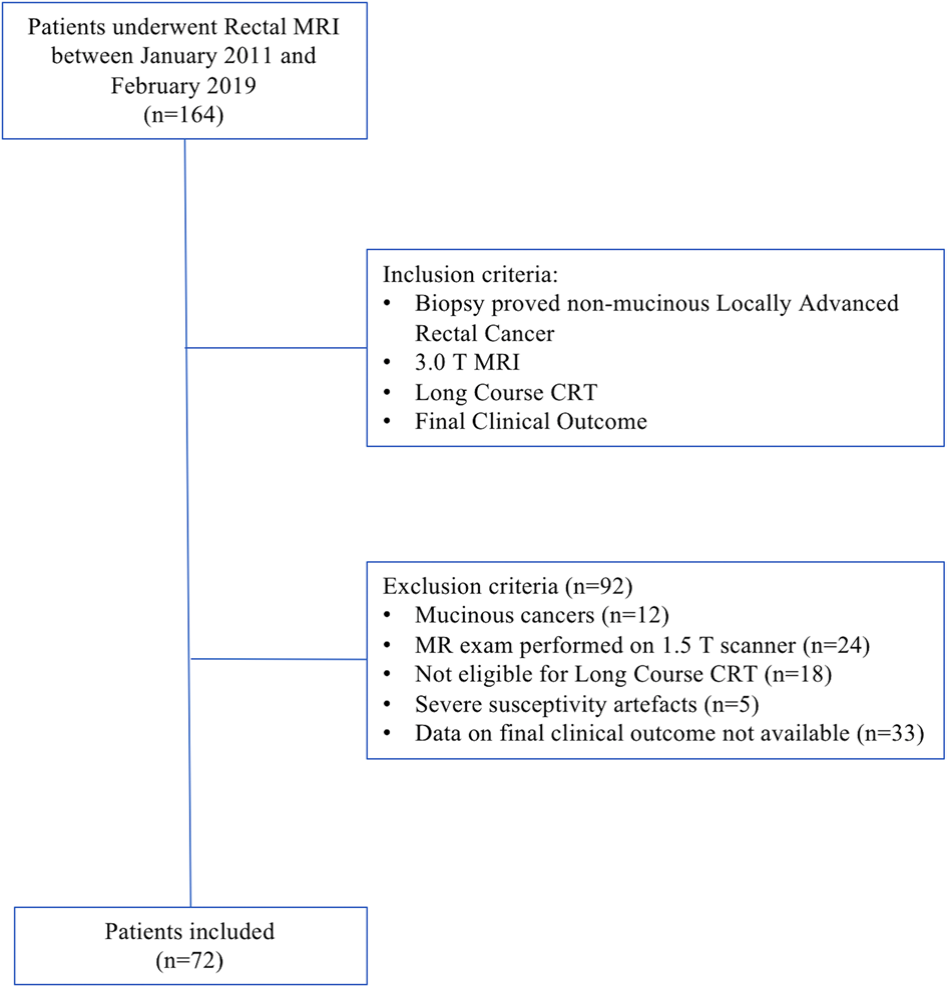
Study flowchart.

The study received formal approval from the Ethical Committee of the University G. D’Annunzio of Chieti-Pescara, Italy; informed consent was waived by the same ethics committee that approved the study (Comitato Etico per la Ricerca Biomedica delle Province di Chieti e Pescara e dell’Università degli Studi “G.d’Annunzio” di Chieti e Pescara). The study was conducted according to ethical principles laid down by the latest version of the Declaration of Helsinki. A total of 164 patients who underwent clinically indicated rectal MRI for primary staging between January 2011 and February 2019 at our institution were retrospectively included (Fig. 1). Inclusion criteria were 1) non-mucinous locally advanced rectal cancer confirmed via biopsy, 2) MRI performed using a 3.0 T scanner, 3) availability of final clinical outcome (major pathological response or non-major pathological response), 4) long-course CRT. Ninety-two patients were excluded: 12 were mucinous cancers, 24 because the MRI exam was performed on a 1.5 T scanner, 33 were transferred in other institutes and we had no information regarding the final clinical outcome. Moreover, 18 patients were considered unfit for long-course CRT because of their clinical fragility and 5 patients had severe susceptibility artifacts in the pelvis (hip replacement). The final study population was composed of 72 patients.

### Chemoradiotherapy

Radiotherapy was performed by 3D conformal technique, with a total dose of 45 Gy (1.8 Gy/day) to the pelvic nodes, followed by a sequential boost of 5.4 Gy (1.8 Gy/day; total dose 50.40 Gy), or a concomitant boost of 10 Gy (1 Gy/day for 2 times/week; total dose 55 Gy) initially with a 3D-CRT technique and then with simultaneous integrated boost with intensity-modulated radiotherapy (2.2 Gy/day, total dose 55 Gy). Different schedules of drugs including 5-Fluorouracil and leucovorin or capecitabine were administered as concomitant chemotherapy.

### MRI protocol

All patients in this cohort underwent clinically indicated rectal MRI consisting of a standard T2w and a diffusion weighted imaging (DWI) acquisition performed using a state-of-the-art 3 T MR scanner (Achieva, Philips Medical System, Best, the Netherlands) equipped with a phased array surface coil. Both T2w and DWI sequences were axially oriented perpendicular to the tumor major axis defined on a sagittal scan. Patients without contraindications received 20 mg of scopolamine butylbromide (Buscopan, Boehringer Ingelheim, Ingelheim am Rhein, Germany) intravenously to reduce bowel motility^7^. Detailed information regarding the parameters of the MRI protocol are described in Table 1.

**Table 1:** MRI protocol parameters.

	T2-weighted Turbo spin echo*
Repetition time (msec)	3000–5000
Echo time (msec)	80
Section thickness (mm)	3
Section gap (mm)	0
Acquisition Matrix Size	188 × 167
No. of signals acquired	2
Field of view (mm)	150 × 150
Sensitivity Encoding (SENSE)	Yes
Acquisition Time (min)	2.39
No. of sections	30

*MRI parameters are referred to axial T2-weighted images.

### Imaging analysis

**Figure 2:**
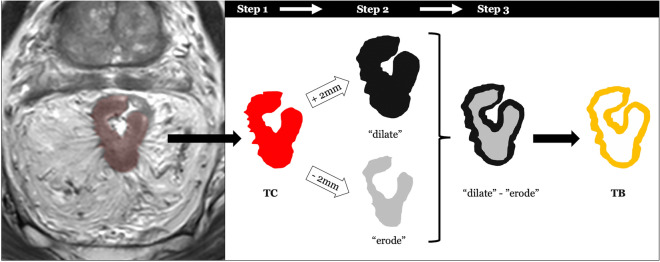
The segmentation process was as follows: in the first step, the whole tumor was manually segmented on axial T2w images and labeled as tumor core (TC). In the second step, the edge of TC was dilated (“dilate”) and eroded (“erode”) by 2 mm, respectively. In the final step, we overlapped the dilated and eroded masks, and subsequently subtracted them to obtain the tumor border (TB). Thus, the TB obtained included the most peripheral portion of the TC and the surrounding tissues.

**Figure 3:**
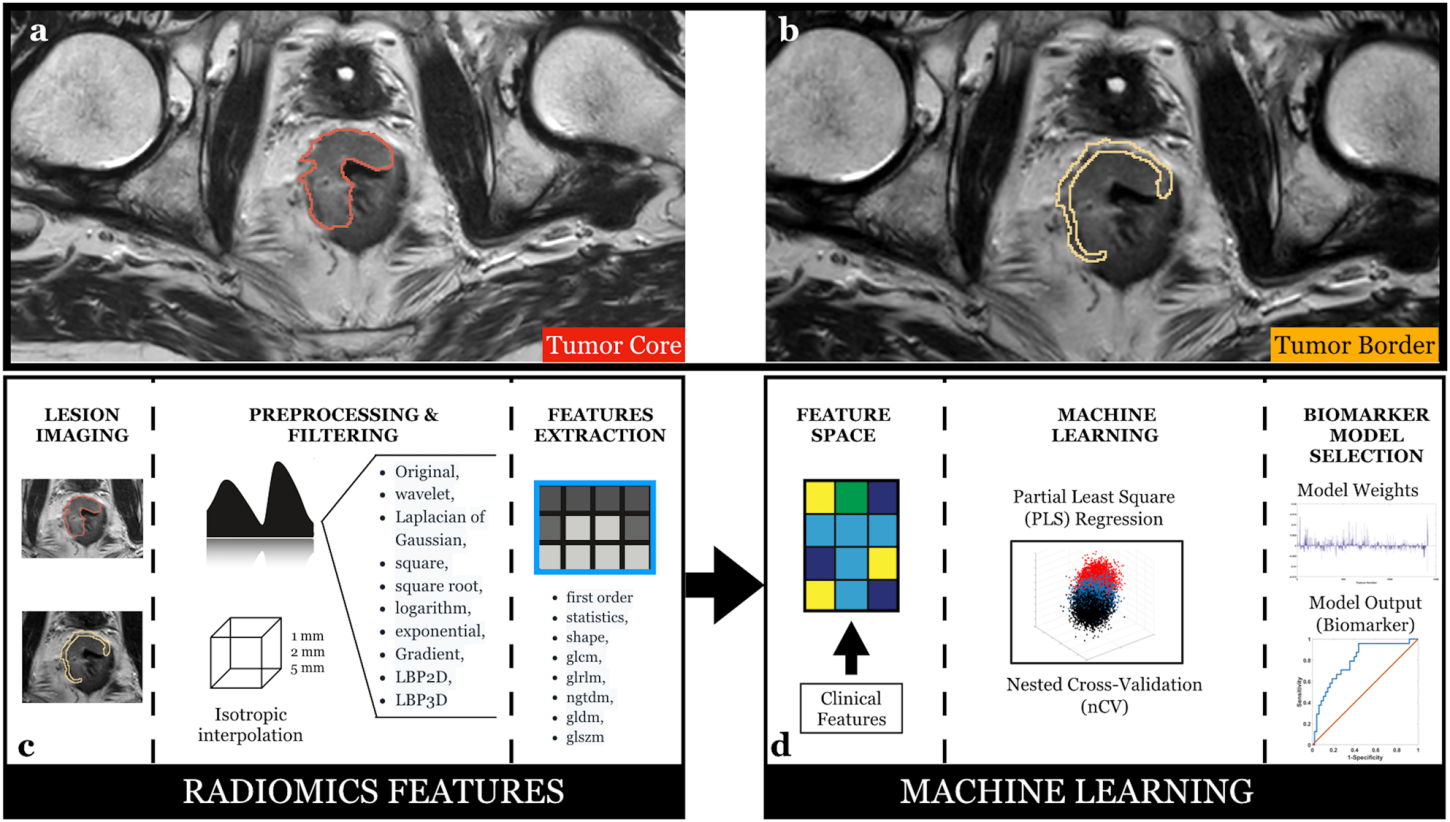
(**a**) Tumor core and (**b**) tumor border on baseline T2-weighted images. (**c**) Schematic representation of the radiomic features extraction process and (**d**) the machine learning approach.

Whole-volume tumor manual segmentation representing the tumor core (TC) was performed on T2w images for each patient by two independent readers with different degrees of experience in abdominal radiology (a radiologist with 5 years of expertise in rectal MRI and a senior resident). All the segmentations were performed on T2w images of the staging MRI and were used as masks for following analysis. The software used for the segmentation was an open-source medical image computing platform, 3DSlicer Version 4.8 (www.3dslicer.org). To create the “border” segmentations (tumor border, TB), a “3dmask_tool” (AFNI) was used^32^. Firstly, both a 2 mm dilatation (“dilate”) and a 2 mm erosion (“erode”) were obtained from the TC of each patient. Secondly, the two masks were subtracted (“dilate”–“erode”) in order to obtain the TB which was 4 mm thick (Fig. 2). All the “border” masks were then checked by the two readers. If necessary, the segmentation was manually corrected in order to include only the outer border of the tumor and adjacent perivisceral fat. TC and TB were shown in Fig. 3a,b.

### Clinical features

Four weeks after the end of the manual segmentation, the two readers in consensus evaluated, for each patient, nine MRI-based clinical features, namely, tumor location (high = 1, middle = 2, low rectum = 3), whole tumor volume (mm^3^), cranio-caudal extension (mm), distance from the internal anal sphincter (mm), mesorectal fascia infiltration (absent = 0, present = 1), extramural vascular invasion (absent = 0, present = 1), extramural depth of invasion (mm), T-stage (1–4) and N-stage (1–2). These MRI-based features were defined “clinical features” and were used in the machine learning model.

### Radiomic features extraction

The extraction of the radiomic features from the masked (TB and TC) T2w images was performed using PyRadiomics, a flexible open-source platform capable of extracting a large panel of engineered features from medical images; this radiomic quantification platform enables the standardization of both feature definitions and image processing^33^. The reproducibility assessment of the features extracted by the two readers from the segmentations of all patients was performed. To avoid data heterogeneity bias and to promote reproducibility, the T2w images and masks were resampled using 3 isotropic voxel dimensions (1 × 1 × 1 mm, 2 × 2 × 2 mm, 5 × 5 × 5 mm). For each segmentation and for each image resolution (1 mm, 2 mm and 5 mm) ten built-in filters (Original, wavelet, Laplacian of Gaussian (LoG), square, square root, logarithm, exponential, Gradient, LBP2D, LBP3D) were applied and seven feature classes (first order statistics, shape descriptors, glcm, glrlm, ngtdm, gldm, glszm) were calculated, which resulted in a total of 1688 radiomic features (Fig. 3c).

### Ethical statement

This study was approved by the local ethics committee. The study used only pre-existing medical data, therefore patient consent was waived.

## Machine learning: partial least square (PLS) regression

Machine learning approaches (also defined multivariate approaches) exploits data multidimensionality to extract useful information. The added value of such approaches is that they identify statistical dependences among variables that are not visible to standard univariate analysis.

However, many radiomic features tend to identify similar image characteristics, generally providing highly redundant information^34^. This means that, when trying to predict an output based on these features, this information redundancy, coupled with the large numerosity of features with respect to samples (e.g. subjects), corrupts the results making the prediction unstable to noise and prone to overfitting and poor generalization^35^. To address this problem, in this work, two main approaches were implemented. The first approach to dampen this effect was to reduce the number of used features by selecting only those that were highly repeatable between the two masks (delineated by the two radiologists) used (r > 0.95). The second approach was to implement a machine learning framework based on a linear regression analysis that employed a space dimension reduction procedure, namely the partial least square (PLS) regression^36,37^. The PLS was used to predict the treatment response in patients with LARC at an early stage from the set of clinical and radiomic features. PLS has been extensively proven to be highly effective in reducing overfitting in the presence of collinearity. The underlying assumption of PLS is that the observed data is generated by a system or process which is driven by a small number of latent variables.

PLS allows the construction of regression equations reducing the predictors to a smaller set of uncorrelated components, i.e. a linear combination of the original predictors, and performs regression on these components^36,38^. The goal of PLS is to identify components that capture most of the information in the independent variables (e.g. linear combinations of all radiomic features) that is useful for predicting the dependent variable (e.g. treatment response), while reducing the dimensionality of the regression problem by using fewer components than the original number of independent variables. PLS can be conceived as a supervised learning version of the Principal Component Analysis (PCA)^39,40^. Of note, the learning process (fitting) of the PLS algorithm provides regression loadings that can be used to retrieve the weights (β-weights) linking the original independent variables with the dependent variable, depicting the importance and sign of the original variables in the prediction process. The PLS has one hyperparameter to be optimized, namely the number of uncorrelated components (linear combinations of the original independent variables) to be used in the regression.

In order to perform hyperparameter optimization and evaluate the generalizable performance of the procedure three sets are required, a trained set, where the model is trained as a function of different hyperparameter values, a validation set, where the best hyperparameter is chosen, and a test set, where the final model is tested. However, when the number of samples is small, this separation of data can greatly reduce the training sample, impairing appropriate fitting of the data-driven model.

An extension of this approach that allows to minimize the effect of the loss of samples in the different sets is the nested cross-validation (nCV)^41–43^. In nCV, data are divided in folds and the model is trained on all data except one-fold in an iterative, nested manner. Whereas the outer loop estimates the performances of the model among iterations (test), the inner loop evaluates the optimal hyperparameter (validation). If the number of folds equals the number of samples (one-fold per sample) the procedure is defined leave-one-out nCV^44,45^. This approach is highly suited for medical applications where each sample represents one subject. In this work, a leave-one-out nCV was implemented to optimize the PLS number of components and to assess the PLS generalization performance.

The leave-one-out nCV PLS analysis was repeated multiple times considering standalone clinical features, standalone radiomic features (with an inter-radiologist repeatability of r > 0.95) and combined clinical and radiomic features. MRI-based clinical and radiomic features were also analyzed multiple times considering TC only, TB only, as well as both TC and TB radiomic features (Fig. 3d). The machine learning analyses were implemented in Matlab.

### Reference standard

The major pathologic response, assessed for 69 patients on surgical specimens, was considered to be Tumor Regression Grade (TRG) 1 or 2 scores according to Mandard’s classification^46–48^. Alternatively, for 3 patients, a sustained complete clinical response with repeated negative MRI examinations and endoscopy with or without biopsy was considered surrogate for a major pathological response for patients enrolled in watch-and-wait protocols^49^. More in detail, a sustained complete response was defined as a long-term recurrence-free clinical follow-up (minimum follow-up period of 1 year)^50^.

### Statistical analysis

The classification performances were assessed through Receiver Operating Characteristic (ROC) analyses considering the inferred (out-of-training-sample) treatment response to therapy. Patients who responded to therapy (major pathological response) were attributed to the “negative” group, whereas patient showing a non-major pathological response were attributed to the “positive” group. The ROC analyses were also performed on random shuffled outcomes to simulate the null hypothesis and evaluate its confidence interval (repeated 10^6^ times). The ROC analysis delivered an Area Under the Curve (AUC) which, using the random shuffled outcomes, could be transformed into a z-score for assessing its statistical significance. The Statistical Analysis was performed in Matlab.

## Results

Out of the 72 patients included in the study, 48 were male (67%), and the mean age was 65 (IQR: 57.5–73.8) years. 48 (67%) patients showed a major pathological response and 24 (33%) a non-major pathological response (Table 2).

**Table 2:** Descriptive baseline characteristics of included patients (n = 72).

Variable	Value	
Gender		
Male	48 (67%)	
Female	24 (33%)	
Mean age (IQR*)	65 (57.5–73.8)	
MRI exam	72	
**Clinical MRI assessment**		
Mean cancer volume (Mean ± SD)	21,885 ± 20,539 mm^2^	
Location		
High	7 (10%)	
Middle	33 (46%)	
Low	32 (44%)	
Craniocaudal extension (Mean ± SD)	55 ± 22 mm	
Distance from IAS (Mean ± SD)	31 ± 27 mm	
Depth of extramural invasion (Mean ± SD)	7 ± 7 mm	
Presence of mesorectal fascia infiltration	44 (61%)	
Presence of EMVI	50 (69%)	
Primary cT stage**		
T1-T2	14 (19.4%)	
T3	55 (76.4%)	
T4	3 (4.2%)	
Primary cN stage**		
N0	2 (2.7%)	
N1	22 (30.6%)	
N2	48 (66.7%)	
Treatment response***		
MR	48 (67%)	34 TRG1 (71%)
		14 TRG2 (29%)
nMR	24 (33%)	17 TRG3 (71%)
		7 TRG4 (29%)

*IQR Inter-Quartile Range; SD Standard Deviation; IAS Internal Anal Sphincter; EMVI Extramural Vascular Invasion; MR Major Pathological Response; nMR non-Major Pathological Response.

**Assessed with MRI and derived from clinical MRI reports in the hospital’s patient database.

***Assessed according to Mandard Tumor Regression Grade (TRG) system on surgical specimen after neoadjuvant treatment in 69/72 patients. In three patients, a sustained complete clinical response (with repeated negative MRI examinations and endoscopy with or without biopsy) was considered surrogate for a complete response; the follow-up (mean ± SD) was 47 ± 11 months.

Nine clinical features were available and all of them were considered in the machine learning analysis. Moreover, 1688 radiomic features were extracted for the three image resolutions employed (1 mm, 2 mm and 5 mm) and for TC and TB, leading to a total of 10,128 (1688 × 3 × 2) features. 1405 of these features showed an inter-reader correlation of r > 0.95 and were used for further analysis.

**Figure 4:**
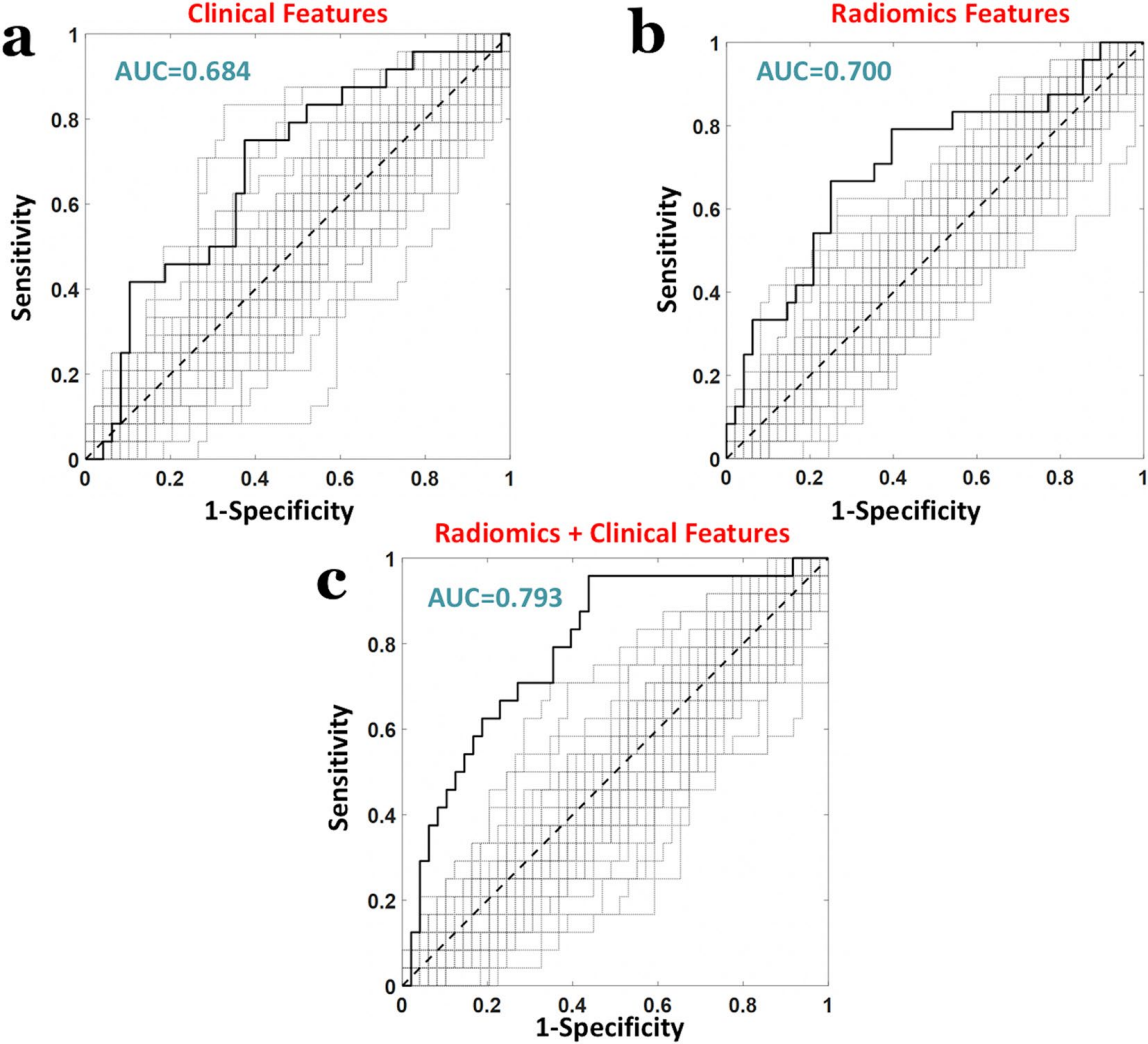
ROC analyses of the machine learning (PLS) classification performance predicting response to therapy (major pathological response as “positive”, non-major pathological response labelled as “negative”) when considering (**a**) standalone clinical features (**b**) standalone radiomic features and (**c**) combined clinical and radiomic features.

When considering the 9 standalone clinical features an AUC = 0.684 was obtained (z = 2.53, *p* = 11 × 10^−3^, Fig. 4a). When employing standalone radiomic features with an r > 0.95, i.e. 1405 radiomic features, an AUC = 0.700 was obtained (z = 2.75, *p* = 5.9 × 10^−3^, Fig. 4b). Importantly, the highest AUC was obtained when combining the 9 clinical features with the 1405 radiomic features, obtaining an AUC = 0.793 (z = 4.00, *p* = 5.6 × 10^−5^, Fig. 4c).

**Figure 5:**
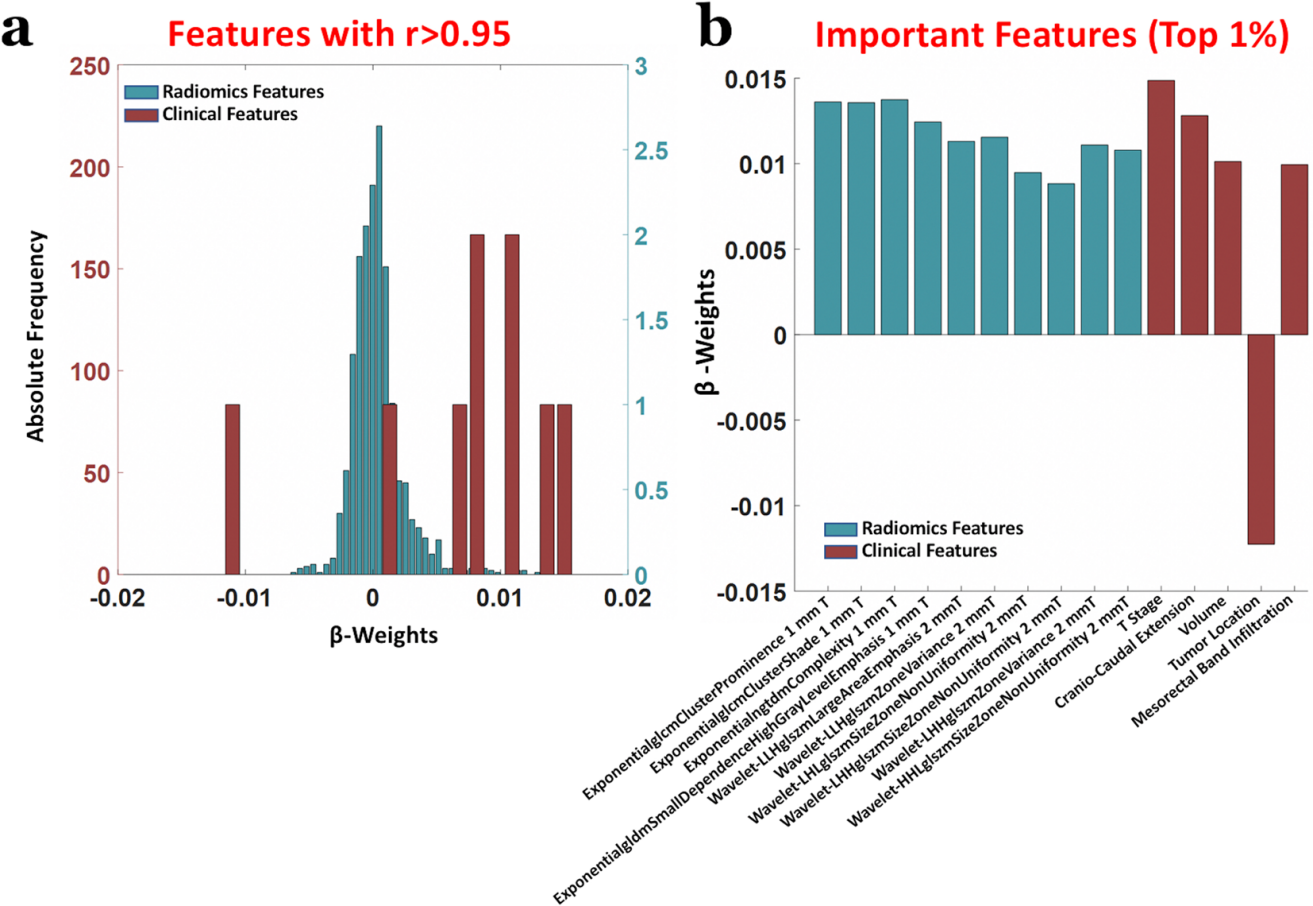
(**a**) Distributions of the PLS β-weights of radiomic and clinical features when the machine learning analysis was performed on combined data. (**b**) β-weights associated to the top 1% of features with the largest β-weights in magnitude, i.e. those most impacting the prediction.

**Figure 6:**
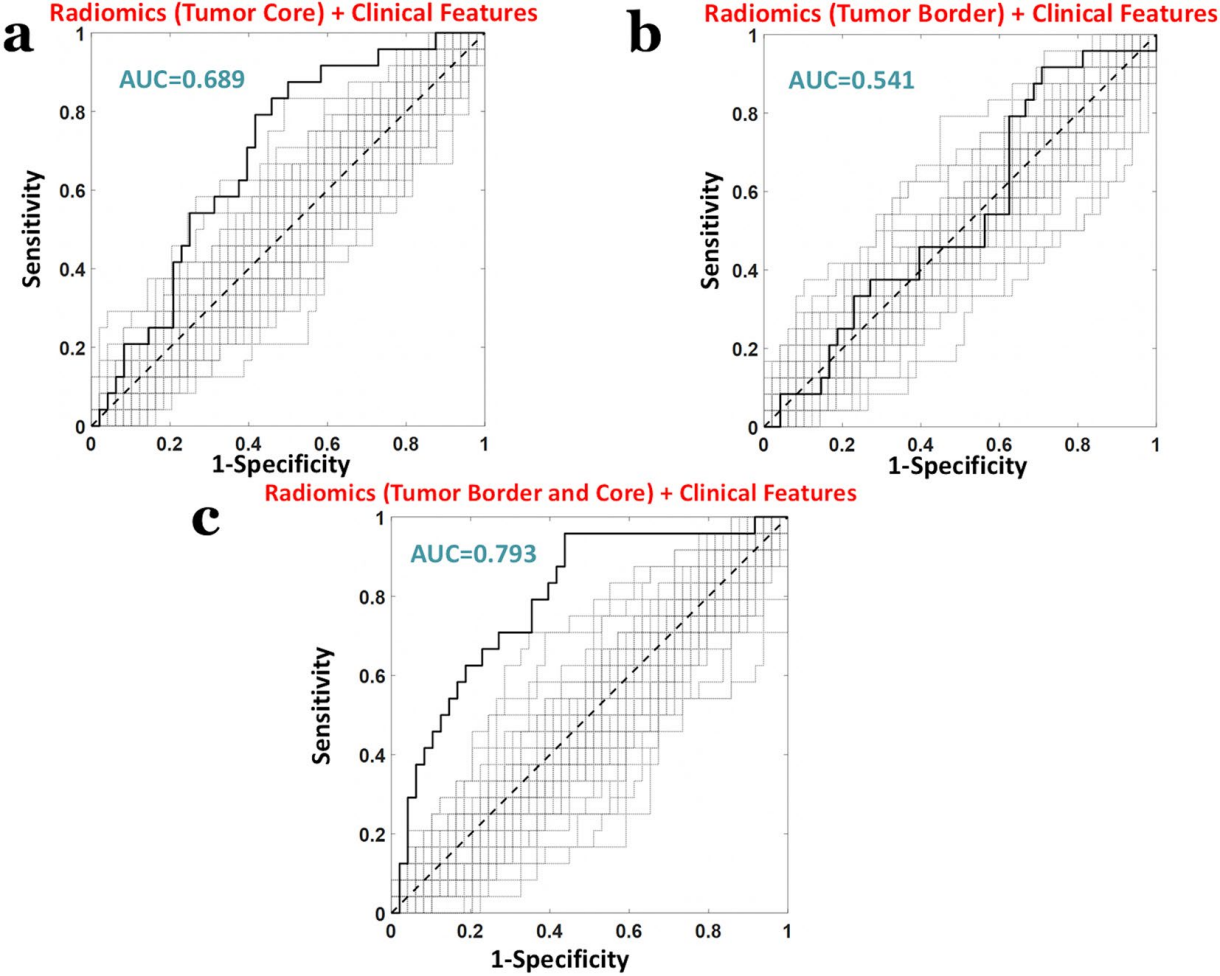
ROC analyses of the machine learning (PLS) classification performance predicting response to therapy (major pathological response labelled as “positive”, non-major pathological response labelled as “negative”) when considering (**a**) clinical and TC radiomic features (**b**) clinical and TB radiomic features and (**c**) clinical and TC + TB radiomic features.

The weights of the PLS (β-weights), when the machinery was trained on both clinical and radiomic features, are shown in Fig. 5. Figure 5a reports the distributions of the β-weights for radiomic and clinical features, whereas Fig. 5b depicts the β-weights associated to the top 1% of features with the largest β-weights in magnitude, i.e. those most impacting the prediction. Importantly, for all except one (tumor location), of the top 1% of features, the weights were positive, that considering the value of “0” attributed to patients with major pathologic response and the value of “1” to the others during the multivariate regression, depicted a worse response to treatment with increasing feature value. Of note, the only important feature with negative weight was tumor location that, considering the labelling value attributed as a function of location (1 = High, 2 = Middle, 3 = Low), delineated a better response to treatment for tumors in the low rectum. Finally, the impact of TC and TB on the results was assessed. When using the 9 clinical features and 790 radiomic features (with an inter-radiologist repeatability of r > 0.95) extracted only from the TC, an AUC = 0.689 was obtained (z = 2.60, *p* = 9.3 × 10^−3^, Fig. 6a). When using the 9 clinical features and 626 radiomic features (with an inter-radiologist repeatability of r > 0.95) extracted only from the TB, an AUC = 0.541 was obtained (z = 0.56, *p* = 0.57, Fig. 6b). Indeed, a highly synergistic effects was obtained when combining TB and TC features, replicating the results previously found with an AUC = 0.793 (z = 4.00, *p* = 5.6 × 10^−5^, Fig. 6c).

## Discussion

Our results demonstrated that an MRI-based machine learning “clinical-radiomic” approach was an accurate method to predict, at an early stage, the treatment response of patient with LARC.

Importantly, the addition of novel radiomic features to standard T2w-based clinical features was indeed useful in the improvement of the prognostic model. Moreover, the prediction further improved when both TC and TB radiomic features were included. These results confirm the promising role of baseline T2w-imaging for the prediction of clinical-outcome^21,22,51,52^. Recent studies have demonstrated the prognostic role of tumor morphology and the adjacent perirectal tissue assessed on T2w-imaging^10,53^. Furthermore, the importance of the perirectal tissue status for the final clinical outcome was also underlined by the significant reduction of the local recurrences with the introduction of TME thanks to the complete excision of microtumors around the cancer^54^. To the best of our knowledge our study, apart for the added value of radiomic features to standard MRI-based clinical metrics, is the first demonstrating the synergic role between feature extracted from the tumor core and the tumor border in the early prediction of response to therapy. Since the tumor border naturally include a certain degree of mesorectal compartment, our results confirm that also perirectal tissues contain useful information for the prediction of treatment response, as recently showed by Shaish et al.^55^.

Other studies based on radiomics were recently proposed in literature. However, different radiomics approaches were used: for example, a delta-radiomics approach was used to analyze the variation of quantitative features over time. In this way, Jeon et al. analyzed the difference in radiomic features before and after CRT. The authors demonstrated that delta radiomics signatures were successful predictors of treatment response and independent prognostic factors^56^. In other studies, the predictivity of more extensive MRI protocols was explored^57,58^. For example, Cui et al. investigated the predictive role of a multiparametric MRI approach including T2w, contrast-enhanced T1w and ADC images^57^. They demonstrated that a radiomics-based nomogram could predict pathological complete response in patients with LARC. In the same way, Liu et al. developed a radiomics model based on T2w images and DWI before and after chemoradiotherapy resulting in 30 selected features showing good discrimination performance^59^. Additionally, Wan et al., recently published a retrospective study including pre and post neoadjuvant MRI examination from which radiomics features were extracted. Their delta-radiomics signature based on T2w images and DWI showed an AUC of 0.91 for the pathologic complete response prediction^60^. Furthermore, Liu et al. recently developed and validated a multiparametric MRI-based radiomics signature including T2w images and ADC for the prediction of distant metastasis in LARC^61^.

Differently from these approaches, our study was focused only on the primary staging and included only T2w images. In fact, our primary aim was to investigate biomarkers of treatment response at an early stage using the most widely used and recommended MRI imaging^7^. Focusing of the important features deemed as such by the machine learning algorithm, in our study the principal predictive features were mainly focused on tumor heterogeneity and clinical, visually assessed, characteristics. For example, tumors with high degree of internal heterogeneity, N+ and with a high clinical T staging were more likely associated to a poor treatment response. Furthermore, cancers located in low rectum were found to have a more favorable outcome thus confirming the crucial role of neoadjuvant chemo-radiation therapy in this category of patients^62^. Since most of the predictive features were extracted by 1–2 mm resolution voxels instead of 5 mm, our results support the recommendations of the last European Society of Gastrointestinal and Abdominal Imaging (ESGAR) consensus meeting^7^. In fact, according to these guidelines, the slice thickness of axial T2-weighted images should be equal or inferior to 3 mm^7^. Similarly to other studies, the accuracy of the prediction improved when a qualitative MRI assessment is added to a quantitative radiomic-based analysis^21,51^. In this way we are confident that the development of other “omics” disciplines may further improve the overall accuracy of the treatment response prediction^63,64^.

Our study has some limitation. First of all, the sample size is limited and our study lacks of a validation cohort. However, the nCV implemented in our study minimized the effect of reduced number of samples and overfitting^42^. Moreover, the limited sample size was largely due to the exclusion of all patients who underwent rectal MRI on a different magnet (1.5 T), patients who underwent a different radiation therapy than long-course, and patients without known final clinical outcome known. Indeed, future studies on larger cohorts are warranted to confirm our findings, and to further improve the classification outcome.

Secondly, ours is a retrospective single-center study. For this reason, further studies, possibly prospective and multicentric need to be warranted to define a potential standardization of our approach.

Third, some patients did not receive scopolamine butylbromide intravenously due to contraindications. In addition, our 3 T MR scanner is routinely scheduled for a software upgrade which might have improved the image quality over time. Nonetheless, these changes did not affect nor modify the main protocol parameters and reflect the daily clinical practice. Fourth, we only focused our prediction on T2-weighted imaging, without considering other MRI techniques, such as DWI and Dynamic Contrast Enhanced Imaging (DCE). However, since our objective was the prediction at the primary staging, the potential role of DWI and DCE at this time point is controversial. In fact, there is no clear added benefit for DWI regarding T, N, MRF and EMVI assessment based on current evidence. On the other hand, DCE-MRI should be considered as a research tool only and not yet ready to be adopted routinely^7^.

## Conclusion

A pre-treatment, MRI-based, “clinical-radiomic” machine-learning model accurately predict the treatment response in patients with locally advanced rectal cancer. The prediction improved when combining MRI-based clinical features and radiomic features, the latter extracted from both tumor core and the tumor border. Exploiting the method, patients with locally advanced rectal cancer could benefit from a tailored approach including conservative strategies. Prospective validation studies in randomized clinical trials are warranted to better define the role of radiomics in the development of rectal cancer precision medicine.

## Data Availability

The datasets generated during and/or analyzed during the current study are not publicly available due to the clinical and confidential nature of the material but can be made available from the corresponding author on reasonable request.
